# Supplementary material for: Modeling hepatitis C micro-elimination among people who inject drugs with direct-acting antivirals in metropolitan Chicago
Source: PLoS One. 2022 Mar 10;17(3):e0264983. doi: 10.1371/journal.pone.0264983 (PMC8912265; doi:10.1371/journal.pone.0264983)
Supplement: S1 Table — (DOCX) [file pone.0264983.s001.docx]

## Modeling hepatitis C micro-elimination among people who inject drugs with direct-acting antivirals in metropolitan Chicago

## Supplemental Materials

**S1 Table. Parameters for the generation of the synthetic population.**

| **Parameter description** | **Value** | **Range** | **Refs/Notes** |
| --- | --- | --- | --- |
| Probability of chronic infection | 0.67 | 0.49-0.74 | (1, 2) |
| Attrition rate (per year) | 0.024 | 0.01-0.08 | (3) |
| Burn in days | 365 | - | Calibrated by observing the time necessary for the HCV incidence to stabilize. |
| Initial PWID population | 32,000 | 30,000-34,000 | (4) |
| Mean injection career duration (years) | 30.3 | 10-35 | (3) |
| Probability of cessation | 0.232 | 0.13-0.33 | (3) |
| Probability of acute HCV-infected PWID at time of inclusion | 0.05 | 0.01-0.09 | 0.9% prevalence among newer PWID, and 9% among other* PWID (5) |

*PWID who acquired HCV prior to initiating into injection drug use through other modes (e.g. sharing non-injection drug paraphernalia such as snorting straws.

References

1. Boodram B, Hershow RC, Cotler SJ, Ouellet LJ. Chronic hepatitis C virus infection and increases in viral load in a prospective cohort of young, HIV-uninfected injection drug users. Drug Alcohol Depend 2011;119:166-171.

2. Dahari H, Feinstone SM, Major ME. Meta-analysis of hepatitis C virus vaccine efficacy in chimpanzees indicates an importance for structural proteins. Gastroenterology 2010;139:965-974.

3. Hser Y-i, Hoffman V, Grella CE, Anglin MD. A 33-year follow-up of narcotics addicts. Archives of General Psychiatry 2001;58:503-508.

4. Tempalski B, Pouget ER, Cleland CM, Brady JE, Cooper HL, Hall HI, Lansky A, et al. Trends in the population prevalence of people who inject drugs in US metropolitan areas 1992–2007. PloS one 2013;8:e64789.

5. Mackesy-Amiti ME, Boodram B, Williams C, Ouellet LJ, Broz D. Sexual risk behavior associated with transition to injection among young non-injecting heroin users. AIDS Behav 2013;17:2459-2466.
