# Supplementary material for: Modeling hepatitis C micro-elimination among people who inject drugs with direct-acting antivirals in metropolitan Chicago
Source: PLoS One. 2022 Mar 10;17(3):e0264983. doi: 10.1371/journal.pone.0264983 (PMC8912265; doi:10.1371/journal.pone.0264983)
Supplement: S2 Table — (DOCX) [file pone.0264983.s002.docx]

## Modeling hepatitis C micro-elimination among people who inject drugs with direct-acting antivirals in metropolitan Chicago

**S2 Table. HCV model infection parameters.**

| **Description** | **Value** | **Range** | **Refs** |
| --- | --- | --- | --- |
| Duration of acute infection in a naïve individual | 102 | 77-127 | (1, 2 ) |
| Duration of acute infection in a recovered individual | 28 | 8-48 | (2 ) |
| Time taken for an infected individual to become infectious | 3 | 2-4 | (2 ) |
| Probability of a recovered individual clearing virus upon re-exposure | 0.85 | 0.75-0.95 | (2, 3) |
| Probability of spontaneous viral clearance upon first exposure- females | 0.346 | 0.30-0.40 | (4) |
| Probability of spontaneous viral clearance upon first exposure - males | 0.121 | 0.10-0.14 | (4) |
| Probability that a naive PWID will be infected in a receptive sharing event with an infected PWID | 0.01 | 0.0005-0.05 | (5, 6) |

References

1. Dahari H, Major M, Zhang X, Mihalik K, Rice CM, Perelson AS, Feinstone SM, et al. Mathematical modeling of primary hepatitis C infection: noncytolytic clearance and early blockage of virion production. Gastroenterology 2005;128:1056-1066.

2. Dahari H, Feinstone SM, Major ME. Meta-analysis of hepatitis C virus vaccine efficacy in chimpanzees indicates an importance for structural proteins. Gastroenterology 2010;139:965-974.

3. Osburn WO, Fisher BE, Dowd KA, Urban G, Liu L, Ray SC, Thomas DL, et al. Spontaneous control of primary hepatitis C virus infection and immunity against persistent reinfection. Gastroenterology 2010;138:315-324.

4. Micallef J, Kaldor J, Dore G. Spontaneous viral clearance following acute hepatitis C infection: a systematic review of longitudinal studies. Journal of Viral Hepatitis 2006;13:34-41.

5. Vickerman P, Hickman M, Judd A. Modelling the impact on Hepatitis C transmission of reducing syringe sharing: London case study. Int J Epidemiol 2007;36:396-405.

6. Rolls DA, Daraganova G, Sacks-Davis R, Hellard M, Jenkinson R, McBryde E, Pattison PE, et al. Modelling hepatitis C transmission over a social network of injecting drug users. Journal of Theoretical Biology 2012;297:73-87.
